# The Continued Use of Social Commerce Platforms and Psychological Anxiety—The Roles of Influencers, Informational Incentives and FoMO

**DOI:** 10.3390/ijerph182212254

**Published:** 2021-11-22

**Authors:** Jinjie Li, Jiayin Qi, Lianren Wu, Nan Shi, Xu Li, Yuxin Zhang, Yinyin Zheng

**Affiliations:** 1 School of Tourism Management, Shanghai Normal University, Shanghai 201418, China; leejinjie@shnu.edu.cn; 2 Institute of Artificial Intelligence and Change Management, Shanghai University of International Business and Economics, Shanghai 200336, China; qijiayin@139.com (J.Q.); yuxin5966@163.com (Y.Z.); 3 Key Lab of Data Science and Management Decision, Shanghai University of International Business and Economics, Shanghai 200336, China; 4 Key Laboratory of Trustworthy Distributed Computing and Service, Ministry of Education, Beijing 100086, China; 5 School of Management, Shanghai University of International Business and Economics, Shanghai 200336, China; shinan10@hotmail.com (N.S.); 19328008@suibe.edu.cn (Y.Z.); 6 YunlianZhigao (Beijing) Information Technology Research Institute Co. Ltd., Beijing 100086, China; lixubupt@163.com

**Keywords:** influencer marketing, social commerce, FoMO, mental health, informational incentives, materialism, social engagement, psychological anxiety, compulsive buying

## Abstract

Why does the continued use of social commerce platforms fail to promote consumer wellbeing? This study explores the roles of influencers, informational incentives and fear of missing out (FoMO) in the relationships between social commerce platform use and consumer mental health. Data were obtained through questionnaires, as well as constructing a research model. Statistical analysis and path analysis of the structural equation model were performed by the software IBM SPSS and AMOS, and the following results were obtained. (1) Influencer expertise and interactivity, informational incentives and FoMO have a significant impact on consumers’ continued use of social commerce platforms. (2) Materialism has no significant effect on consumer social commerce platform use. (3) FoMO mediates the relationships between informational incentives and continued use of social commerce platforms. (4) Consumers’ continuous use of social commerce platforms has a strong relationship with mental health. (5) Continued use of social commerce platforms can lead to intense social engagement, as well as more severe outcomes such as psychological anxiety and compulsive buying. The findings of the paper have important implications for the development of social business theory and management practice.

## 1. Introduction

With the development of social networking sites (SNSs), mobile e-commerce and mobile payment technologies, social commerce has gained a new round of rapid renewal, and more emerging social commerce platforms have sprung up. Social commerce refers to the phenomenon of applying socialized elements such as attention, sharing, communication, discussion and interaction to the e-commerce transaction process. Social commerce platforms refer to apps and websites that support social commerce activities, such as Xiaohongshu, Mogujie, Kwaishop, Douyinec, etc., which developed in the Chinese context. There are similar social commerce platforms in other countries around the world, such as Amazon Shopping, OfferUp, Instagram, Facebook, and so on.

Specifically, from the perspective of consumers, social commerce is reflected in both the selection of stores and comparison of products before purchase, communication and interaction with social media influencers (SMIs) and e-commerce enterprises through social commerce platforms, and evaluation and sharing of experiences after purchase. From the perspective of e-commerce enterprises, through the web 2.0 applications and cooperation with social media influencers, they can complete the marketing, promotion and final sales of their products. Social commerce has two core features: first, it has the role of a shopping guide; second, there is interaction and sharing between users or between users and enterprises.

Research on social commerce emerged at the same time as business practices, and academic research on social commerce received extensive attention from scholars at the early stage [1,2,3,4]. For example, Zheng et al. (2013) developed a semi-supervised system (ORQM) for estimating the quality of online reviews in social commerce and found that social features contribute most to the system [5]. Kim et al. (2013) proposed the major characteristics of social commerce and emphasized that trust directly affects two variables (purchase and word-of-mouth intentions) of trust performance [6]. Hajli (2015) suggested that consumers use social commerce to interact and generate content, which in turn increases perceived trust and purchase intent [7].

With the enhancement of the integration of social networking sites and e-commerce and the emergence of new social commerce platforms, academic research in the context of social commerce has gained a lot of new research results [8,9,10,11,12]. For example, Chen et al. (2015) investigated the decision-making mechanisms of consumers in a social commerce context [13]. Shanmugam et al. (2016) and Doha et al. (2019) studied the application of social commerce structures [14,15]. Lee et al. (2015) and Li et al. (2018) studied the role of likes (thumbs-up) in the context of social e-commerce [16,17]. In addition, trust has been widely studied as an important feature in social commerce [18,19,20,21].

The continueduse of social commerce platforms, like the use of traditional social media and smart-phones, has brought convenience to consumers in shopping but also brought various problems to consumers’ life and learning, such as anxiety, depression, distraction, insomnia and compulsive buying [22]. Continued use of social commerce platforms in this study refers to consumers increasing or maintaining the frequency of their previous use, as well as continuing to use their current social commerce platforms.

According to the literature, it was found that there is less research on the impact of the continued use of social commerce platforms on consumers’ lives, learning and mental health. There is insufficient research on the antecedents and consequences of the continued use of social commerce. Therefore, there is an urgent need to explore this issue in the current social commerce field. We mainly discuss the impact of the use of social commerce platforms from FoMO, psychological anxiety and compulsive buying, and conduct a review of existing related research.

This study empirically examines the antecedents and consequences of consumers’ continued use of social commerce platforms. The theoretical value of this work is to fill the gap in research on relationships between the continued use of social commerce platforms and consumers’ psychological wellbeing. Moreover, it has important implications for marketing practices and customer management on social commerce platforms.

## 2. Literature Review

### 2.1. SSO Framework

The SSO theoretical framework was first proposed by Koeske et al. and applied to study the relationship between managers’ mental health and professional performance [23]. Subsequently, the theoretical framework has been widely used [24,25], including being applied to the use of technology [26]. The SSO theoretical framework consists of three main components: stressor(s), strain and outcome(s).

In this paper, we use the SSO framework to analyze the antecedents and consequences of social commerce platform use and consumers’ mental health. The stressors of continuous use of social commerce platforms are social media influencers (expertise/interactivity), informational incentives (information promotions/discount) and personal trait (materialism). The strains are fear of missing out and continued use of social commerce platforms. The outcomes are social engagement, social support, psychological anxiety and compulsive buying.

### 2.2. Fear of Missing Out

With the rapid development of social APPs/websites, such as WeChat, Twitter, Tik Tok, etc., dependence on social media websites and mobile apps has become a common phenomenon, leading to the popularity of a new term: “fear of missing out”. Fear of missing out (FoMO) is defined as “a pervasive apprehension that others might be having rewarding experiences from which one is absent” [27]. FoMO is a consequence of social media use [28], social network site use [29,30] and problematic smart-phone use [31,32]. In the past decade, FoMO has received extensive attention from scholars all over the world, and many important research results have been achieved. As shown in Table 1, the representative literature of FoMO-related research is given.

### 2.3. FoMO in Marketing

Psychologists generally treat FoMO as an enduring psychological phenomenon independent of context-specific influences [27]. However, to better understand its influence on consumer behavior, FoMO should be defined as a context-specific anxiety. In recent years, FoMO has received widespread attention from marketing scholars and practitioners. As shown in Table 2, the main literature of FoMO-related marketing research is given.

## 3. Research Hypothesis and Model

### 3.1. Influencer Traits

There are many factors that contribute to consumers’ use of social commerce platforms. From the perspective of social media influencers two factors, expertise and interactivity, are important. An influencer’s expertise is the relevant knowledge, experience or skills that the influencer possesses and conveys to his or her followers [6]. Social media influencers demonstrate their expertise in various ways to attract consumers to use social media applications and engage in interaction [50]. For example, in hotel social media marketing, the influencers are some media people with expertise in hotels [51]. Lou and Yuan (2019) stated that an influencer is an individual with expertise in a specific field who develops a significant number of followers who are of marketing value to the brand by regularly posting valuable content on social media [52]. Ki and Kim (2019) indicated that an influencer is an individual who builds credibility among followers based on knowledge of a specific topic and professional qualities [53]. All of the above literature suggests that influencers in social media need to have specialized knowledge.

Another factor that influences consumers’ use of social commerce platforms is interactivity [54]. Xue et al. (2020) state that immediate interaction positively affects perceived usefulness and negatively affects perceived risk and psychological distance, and facilitates social commerce engagement [55].

Therefore, we propose the following Hypothesis H1.

**Hypothesis** **(H1).** *Influencer expertise and interactivity are positively correlated with consumers’ continued use of social commerce platforms*.

### 3.2. Informational Incentives

In this study, information incentives refer to promotion and discount information provided by social commerce platforms, and product experience information shared by platform influencers.

Social commerce platforms will entice consumers to use and shop through information incentives such as numerous advertisements and promotions [16,56]. When studying the Alibaba Double Eleven Shopping Festival, Xu et al. (2017) pointed out that an informational incentive is perceived as enhancing the individual’s knowledge about the activities and is inherently conducive to the maximization of people’s perceived values regarding behaviors [57]. Promotional information refers to a variety of marketing information to advertise activities and products. Such information usually entices and motivates potential consumers to collectively join the shopping activities [58,59]. Accordingly, we propose Hypothesis H2.

**Hypothesis** **(H2).** *Information incentives are positively correlated with consumers’ continued use of social commerce platforms*.

### 3.3. Fear of Missing Out

Fear of missing out (FoMO) leads to higher intensity and willingness of users to use social media sites or mobile apps. For example, Roberts et al. (2020) found that FoMO had a significant effect on social media intensity and social connection [40]. Elhai et al. (2020) also revealed that FoMO was related to social and problematic smart-phone use (PSU) severity [60]. Beyens et al. suggested that adolescents’ FoMO is positively associated with perceived stress related to Facebook use [34].

Meanwhile, FoMO is a common phenomenon in the current mobile internet era. In the context of social media marketing and influencer marketing, FoMO is widely used in marketing [43]. Thus, advertising content in social media that appeals to missing-out anxiety leads to higher intensity social media use [61]. Weideinger et al. (2021) investigated whether adding FOMO messages to Facebook ads improved consumers’ recall of the ads. The results found that consumers performed better in both recall and recognition of ads when FOMO content was present [62]. Karimkhan et al. (2021) found that FoMO had a significant impact on consumer impulsive buying behavior and was also strongly correlated with collectivism, ethnic identity and culture [48]. The presence of FoMO will eventually generate a higher probability of purchasing behavior. For example, Good et al. (2020 and 2021) studied the direct and indirect effects of FoMO appeals on purchase likelihood, and suggested that FoMO-laden appeals can influence consumers’ purchase intentions [46,47]. Based on the above literature, we propose the following Hypotheses H3 and H4.

**Hypothesis** **(H3).** *FoMO positively affects consumers’ continued use of social commerce platforms*.

**Hypothesis** **(H4).** *FoMO mediates the relationships between informational incentives and continuous use of social commerce platforms*.

### 3.4. Materialism

According to existing research literature, the authors found that the internal factors of individuals who use social e-commerce platforms mainly include individual negative emotions and individual materialistic tendencies [63,64]. Among them, individual negative emotions have been extensively studied in the use of social media, and a more consistent research conclusion has been obtained. In the context of purchasing luxury goods, there is related research on the tendency of individual materialism [65,66]. However, in the context of social e-commerce, and when buying daily necessities and common products, will the tendency of individual materialism have an impact and will that impact be exacerbated by information incentives? At present, there is still a lack of research on the above issues. Therefore, this study will explore the influence of individual materialism on the continuous use of social e-commerce platforms, and the follow-up results.

Building off Rokeach’s conceptualization, Mick (1996) defines materialism as a “set of centrally held beliefs about the importance of possessions in one’s life” [67]. Burroughs et al. (2002) examined the relationship between high levels of materialism and subjective well-being and found that conflicting values can lead to psychological tension, which can reduce well-being [68]. Analysis of questionnaires from 204 adults by Fitzmaurice et al. reveals that materialism is significantly correlated with social consumption motives and opinion leadership [69]. Lee et al. (2021) analyzed consumers’ motivations for following SMIs on Instagram from a consumer social psychology perspective, and found that the four motivations for following influencers were authenticity, consumerism, creative inspiration and envy [51]. Therefore, we propose the following Hypothesis H5.

**Hypothesis** **(H5).** *Materialism is positively associated with consumers’ continued use of social commerce platforms*.

### 3.5. Social Engagement

The persuasion of social media influencers and the informational incentives of social commerce platforms are aimed at getting consumers to engage and buy [70,71]. Social commerce platforms provide consumers with valuable information and facilitate consumer information sharing [72]. Tajvidi et al. (2020) studied the relationship between consumer content production and value co-creation and found that information sharing, social support and relationship quality were significantly correlated with corporate brand value creation [73]. Gvili et al. (2021) investigated the relationship between online shopping culture and consumer experience and brand information sharing in the context of social commerce, and the findings reveal that consumer experience is indirectly related to brand information sharing [74].

In addition, social media influencers interact with consumers through social commerce platforms to enhance consumer social engagement. For example, Wang (2020) proposed a model and employed datasets obtained from Douban.com to empirically investigate informational support on consumers’ engagement, and revealed that the two sub-dimensions of social supportive information are positively related to consumer involvement [75].

Based on the above literature, we propose the following Hypotheses H6 and H7.

**Hypothesis** **(H6).** *The continued use of social commerce platforms positively influences consumer social engagement*.

**Hypothesis** **(H7).** *The continued use of social commerce platforms positively affects consumers’ access to information support*.

### 3.6. Psychological Anxiety

It is common knowledge that inappropriate use of social media applications and smart-phones can bring about mental health problems.For example, Dhir et al. (2018) used the SSO theoretical framework to investigate the relationship between social media fatigue and psychosocial well-being, revealing that compulsive media use has a significant effect on social media fatigue and exacerbates user anxiety and depression [37]. Chai et al. (2019) explored the relationship between SNS use and subjective well-being and found that social overload, FoMO and SNS use had significant effects on subjective well-being, with FoMO and social overload acting as moderating variables that moderated the relationship between SNS use and subjective well-being [38]. Rogers et al. (2019) revealed that FoMO and telepressure have also been linked to negative health outcomes among university students, including poor sleep hygiene [76]. Buglass (2017) pointed out that increased SNS use will be positively associated with increased FoMO, and FoMO will mediate the relationship between SNS use and psychological wellbeing [29]. Therefore, we propose Hypothesis H8.

**Hypothesis** **(H8).** *The continued use of social commerce platforms results in higher psychological anxiety*.

### 3.7. Compulsive Buying

The social commerce platform brings a different way of shopping and a new shopping experience to consumers. Studies have shown that in the context of social commerce, consumers are prone to impulsive [77,78] and compulsive [79] buying behaviors owing to the social interactions and informational incentives.

Impulse buying is a quick, unplanned purchase behavior that occurs mainly as a result of simple processing of promotional or advertising messages [80,81,82]. Chen et al. (2016) explored the effect of advertising on consumers’ impulse purchases in Facebook, and their findings revealed that information quality and individual impulse traits have significant effects on consumers’ impulsive purchase behavior [83]. Akram et al. (2018) suggested that situational factors positively influence the online impulse buying among Chinese online shoppers in a social commerce environment [84]. Zafar et al. (2019) emphasized that social media celebrities’ posts and contextual interaction have a significant impact on impulse buying [85]. Hu et al. (2019) revealed that peer influence has an important influence on consumers’ impulsive consumption behavior in a social business environment [86].

Another concept similar to impulse buying but essentially different is compulsive buying, which refers to a bad habit behavior that is a reaction to a bad mood or negative event. Compulsive buying is characterized by the ability to obtain pleasure from the purchase or to relieve negative emotions [87,88,89]. Jin et al. (2020) studied the effects of envy, parasocial interaction and consumer traits on purchase behavior in the context of social commerce, and the experimental results reveal that these factors have a direct or indirect relationship on the outcome of consumer behavior [90]. Focusing on compulsive buyers, Kukar-Kinney et al. (2016) investigated the influence of psychological motivation and context on their behavior, and the results reveal that the shopping environment of daily deal websites is highly seductive to compulsive buyers [91]. He et al. indicated that the high prevalence of compulsive buying in China may be associated with face consciousness, and new online compulsive buying drivers in China include observed buying, daydreaming and emotion [92].

According to the above literature, we propose Hypothesis H9.

**Hypothesis** **(H9).** *The continued use of social commerce platforms is positively associated with consumers’ compulsive buying*.

**Figure 1:**
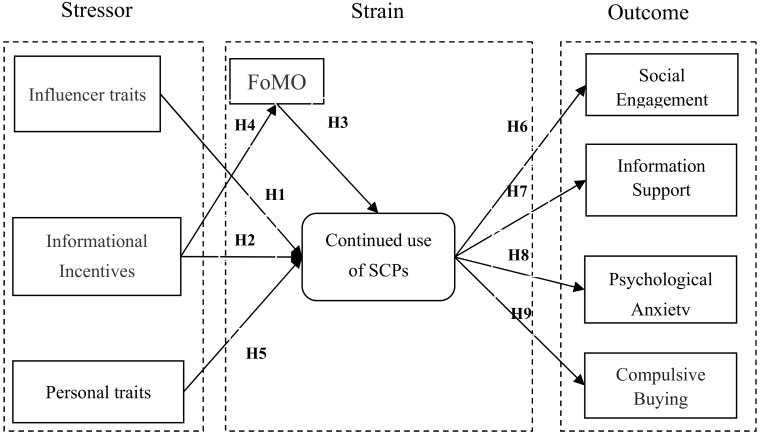
Research Model.

Based on the above hypotheses, the proposed model is summarized in Figure 1.

## 4. Research Design

### 4.1. Data Collection

This research used questionnaires to collect data. The subjects of the survey were users who have used social commerce platforms. The process of issuing and collecting questionnaires was mainly divided into two stages.

The first stage: from 12 May 2021 to 31 May 2021, this stage mainly conducted a pre-survey, and changes and amendments to the questionnaire items, thereby forming a formal questionnaire.

The second stage: 1 June 2021–10 June 2021, this stage involved the formal distribution and collection of questionnaires, through the questionnaire star website (https://www.wjx.cn (accessed on 12 June 2021)), where the respondent filled out the questionnaire.

After the pre-survey, the content of the questionnaire was modified to ensure that the questionnaire met the statistical research standards. After the pre-investigation, the content of the questionnaire was modified to ensure that the questionnaire met the statistical research standards. After the questionnaire was created on the questionnaire star website, it was formally distributed from the questionnaire star. The questionnaire used the Likert 7-point method. As of 10 June 2021, a total of 386 questionnaires had been collected. After removing 20 invalid questionnaires for which respondents took less than 60 s to complete the questionnaire, 366 valid questionnaires were finally obtained. The response rate for questionnaires reached 94.82%.

### 4.2. Descriptive Statistics

The study first used IBM SPSS 25.0 to perform descriptive statistical analysis, correlation analysis, reliability and validity analysis on the questionnaire data. We then used IBM AMOS 25.0 to analyze the path of the research model.

Table 3 summarizes the descriptive information of the dataset. About 32.79% of the respondents were male, and 67.21% were female. As the free survey was conducted in universities, the majority (79.23%) of the respondents were students aged between 20 and 30. About 68.58% of the respondents were undergraduates and 30.87% were postgraduates.

Table 4 shows the variables involved in the study and the results of the correlation analysis.

**Figure 2:**
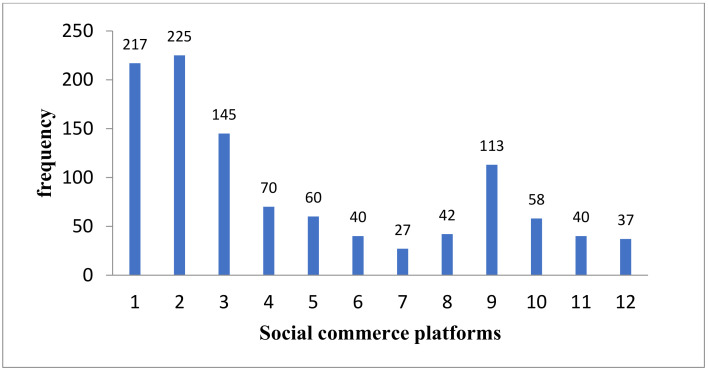
Distribution of social commerce platforms. 1 = Dianping; 2 = Xiaohongshu; 3 = Dewu; 4 = Mogujie; 5 = Meilishuo; 6 = Hers; 7 = Xiaohongchun; 8 = Kwaishop; 9 = Douyinec; 10 = Meiyou; 11 = Fensii; 12 = others.

It can be seen from Figure 2 that in this survey sample, Xiaohongshu is used the most frequently, followed by Dianping, Dewu and Douyinec.

### 4.3. Reliability and Validity Analysis

Reliability analysis is mainly used to evaluate the stability, consistency and accuracy of the scale. There are usually three indicators used to measure reliability, namely the Cronbach alpha coefficient, split-half reliability and test-retest reliability. This study used the Cronbach alpha coefficient to test the stability and accuracy of the scale (see Appendix A, Table A1). The results are shown in Table 5.

Observing the results in Table 5, we can see that the Cronbach coefficients of all latent variables are greater than 0.8, and the Cronbach coefficient of psychological anxiety is 0.931, which exceeds 0.9. This shows that the internal reliability of the questionnaire items is high, and there is high consistency.

In addition, the validity of the questionnaire was analyzed by observing the KMO index and the Bartlett sphere test index. The purpose of this was to determine whether the questionnaire was suitable for factor analysis. When the KMO value is greater than 0.9, it indicates that the questionnaire is very suitable for factor analysis; when it is 0.7–0.9, it indicates that the questionnaire data are suitable for factor analysis. The KMOs of all latent variables in the questionnaire are greater than 0.7, and the *p* values of Bartlett’s sphere test are all 0.000, indicating that the questionnaire is suitable for factor analysis. In addition, the item factor loading coefficients are all above 0.7, and the average variance extracted amounts are all above 0.6.

## 5. Hypothesis Testing

### 5.1. Path Analysis

**Figure 3:**
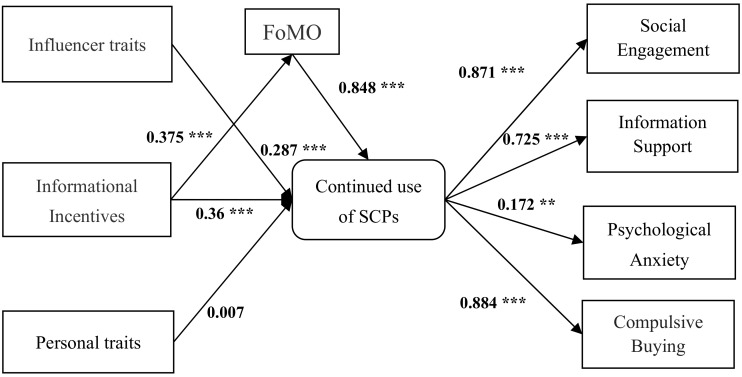
Basic model testing results. *n* = 366; *** *p* ≤ 0.001, ** *p* ≤ 0.01.

We used IBM AMOS 25.0 to analyze the theoretical model. The path coefficients of the model are shown in Figure 3. As seen in Table 6, the fit indices of the model all reach the standard level, which indicates that the model works well.

As can be seen from Table 7, the path coefficient of influencer traits to continue use of the social commerce platform is 0.287, *p* < 0.001, which shows that influencer traits have a significant positive influence on consumers’ continued use of social commerce platforms. Hypothesis H1 is supported.

The coefficient of informational incentives to continue use of social commerce platforms is 0.36, *p* < 0.001, which indicates that informational incentives have a significant positive effect on consumers’ continued use of social commerce platforms. Hypothesis H2 is supported.

The path coefficient of FoMO to continue use of social commerce platforms is 0.848, *p* < 0.001, which suggests that FoMO has a significant impact on consumers’ continued use of social commerce platforms. Hypothesis H3 is supported.

The path coefficient of personal trait (materialism) to continue use of social commerce platforms is 0.007, *p* = 0.779; this indicates that the effect of materialism on consumers’ continued use of social e-commerce platforms is not significant. Hypothesis H5 is not supported.

The path coefficients for continued use of social commerce platforms to social engagement, information support, psychological anxiety and compulsive buying are 0.871 (*p* < 0.001), 0.725 (*p* < 0.001), 0.172 (*p* < 0.05) and 0.884 (*p* < 0.001), respectively. Therefore, Hypotheses H6, H7, H8 and H9 are supported.

### 5.2. Analysis of Mediating Effect

**Figure 4:**
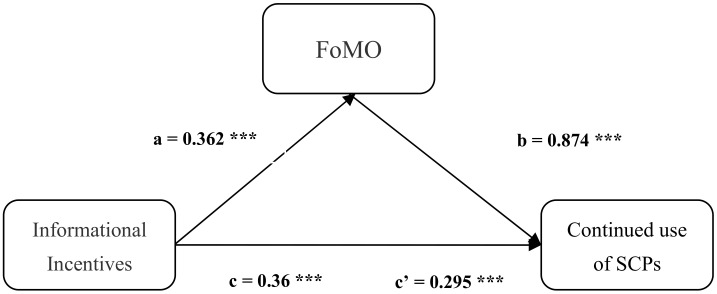
Mediation model. *** *p* < 0.001; *n* = 366; II indicates informational incentives; CUSCP indicates continued use of social commerce platform; FoMO indicates fear of missing out.

In this study, we used Amos 21.0 to analyze the mediating role of FoMO. The mediation model and path coefficients are shown in Figure 4. We found from Table 8 that the coefficients a, b and c’ are significant, indicating that there is a mediating effect of FoMO between informational incentives and continued use of SCPs. Therefore H4 is supported.

## 6. Conclusions

With the rise of live streaming, social commerce platforms have become prevalent in China, such as Xiaohongshu, Kwaishop and Douyinec, which have become the main platforms for entertainment and shopping for Chinese consumers today. This study explored the role of influencers, informational incentives and FoMO in the relationship between the continued use of social commerce platforms and consumer mental health. Our findings are as follows.

(1) Influencer traits (expertise and interactivity), informational incentives and fear of missing out have a significant impact on consumers’ continued use of social commerce platforms.
(2) Materialistic tendencies have no significant effect on consumers’ continued use of social commerce platforms.
(3) FoMO mediates the relationships of informational incentives and continued use of social commerce platforms.
(4) Consumers’ continuous use of social commerce platforms has a strong relationship with mental health. Continued use of social commerce platforms can lead to intense social engagement, as well as more severe outcomes such as psychological anxiety and compulsive buying.

## 7. Discussion and Future Directions

### 7.1. Theoretical Contributions

The social commerce formed by social networks and e-commerce has received extensive attention from scholars since its emergence. After 10 years of development, a series of research results have been achieved. From the available research literature, the areas of social commerce research mainly include the following five aspects.

(1) The differences between e-commerce and social commerce. Specifically, it contains the differences between social commerce and social shopping, as well as the differences between social commerce and e-commerce [93,94]; (2) social commerce types and social commerce technologies [95]; (3) challenges versus benefits [96]; (4) research models of social commerce [97]; (5) social commerce frameworks [98,99].

According to the above, there is a lack of research on the impact of social commerce development on consumers, especially on their mental health. This paper bridges this research gap.

This study collected data through questionnaire surveys and received a total of 366 valid questionnaires. The study used IBM SPSS to perform descriptive statistics, correlation analysis, reliability analysis and validity analysis on the data, and used IBM AMOS to perform a path analysis on the structural equation model proposed in the paper.

The main contribution of this research was to examine the relationship between the continued use of social commerce platforms and consumer mental health in terms of influencers, consumers and information incentives (both from influencers and social commerce platforms). The study extends the scope of current social commerce research.

In addition, fear of missing out was introduced as an independent and mediating variable in the research model. The paper explored the relationship between fear of missing out and the continued use of social commerce platforms. Further, the mediating role of fear of missing out in informational incentives and the continued use of social commerce platforms was investigated. This study extends the scope of the study of fear of missing out.

### 7.2. Practical Contributions

The sudden arrival of COVID-19 has led to the counter-trend development of “social commerce platforms”, making social shopping and community shopping seem to become a trend. There are three reasons for social commerce to explode during the pandemic: low cost, high conversion and value extension.

The findings of this paper provide management insights for social commerce platforms and social media marketing. Specifically, they are as follows. From the perspective of influencers, social commerce platforms should meet consumers’ needs for information sharing and interaction. For example, Xiaohongshu gathered a large number of active consumers who love to shop, and they are always sharing global goodies and shopping tips, guiding many “newbies” who have no online shopping experience to quickly learn how to use the products. They saw the content and shopping tips shared by their predecessors on Xiaohongshu, which made the whole platform become an encyclopedia of online shopping and attract many new consumers.

Social commerce platforms and influencers need to establish a good trust relationship with consumers. Influencers and consumers do not have a transactional relationship with each other, but rather an informational interaction. Through quality platform operations, influencers and consumers are prompted to continuously generate content. When these contents form good word-of-mouth accumulation, consumers will trust the platform and then get involved in business transactions.

From the perspective of informational incentives, the value of social marketing is based on sharing knowledge and content. This is because knowledge and content address the needs of consumers. In the social world, what matters most to consumers is not what you sell, but what consumers need. Xiaohongshu has done a good job at this point, and its path is to conquer consumers with knowledge and content, rather than bombarding them with advertisements.

For example, Xiaohongshu builds a consumer community for the exchange of product and service experience information. By cultivating community influencers, it promotes sharing and community activity. These practices enable consumers to actively search for information in the community instead of passively accepting information. In addition, the user group of social commerce platform is mainly female, and the characteristic of this group is uncertainty to browse products. For this reason, Xiaohongshu does not organize the product display as clearly as other e-commerce platforms, but various types of products will appear on the home page. This move breaks the conventionalized thinking and creates differentiation.

### 7.3. Research Limitations and Future Directions

There are many factors that influence the continued use of social commerce platforms. There are technical aspects of social commerce platforms, such as ease of use, privacy protection and security. These factors have not been discussed in this study. When considering influencer characteristics, this study also focused on the expertise and interactivity of the influencers, and other factors were not discussed. For example, the trust relationships between influencers and consumers are very important factors.

The sustainability of social commerce platforms still faces many issues. These questions are the focus of future research. For example, unlike the strong social relationships constructed between acquaintance social networks, social commerce networks are mainly para-social relationships constructed between strangers [100], and exploring the process of relationship construction between influencers and consumers by introducing communication theories such as para-social interaction and para-social relationships is an important research question [101,102,103]. The issue of trust between consumers and influencers is another important research direction [104,105].

## Figures and Tables

**Table 1:** Representative literature of FoMO-related research.

Author(s)/Year	FoMO as Variable	Variables Related to FoMO	Findings
Przybylski, et al. (2013) [27]	MEV	Individual differences; social media engagement	Demographics, motivations, behaviors, emotions and well-being relate to FoMO.
Alt, D. (2015) [33]	MEV	Academic motivations; social media engagement	FoMO mediates the relationship between social media engagement and motivational factors.
Beyens, I. et al. (2016) [34]	MEV	Need for popularity; need to belong; Facebook use	Teens with high FoMO use Facebook more intensively and are more stressed when they are unpopular on social media.
Oberst, U. et al. (2017) [35]	MEV	Anxiety; depression; social network intensity negative consequences	Both FoMO and SNI mediate the link between psychopathology and negative consequences.
Elhai, J. D. et al. (2018) [36]	IV	Negative affectivity; problematic smartphone use (PSU) severity	FoMO is associated with the severity of problematic smartphone use (PSU).
Dhir, A. et al. (2018) [37]	IV	Depression; anxiety; SNS fatigue; compulsive SNS use	Compulsive media use mediates between FoMO and social media fatigue.
Chai, H. Y. et al. (2019) [38]	MOV	SNS use; social overload; subjective well-being	FoMO moderates the relationship between SNS use and subjective well-being.
Holte, A. J. et al. (2020) [39]	DV	Anxiety; boredom proneness; depression; anxiety attachment	Boredom proneness mediated the relationship of FoMO with anxiety and depression.
Roberts, J. A. et al. (2020) [40]	IV	Social media intensity; social connection; psychological wellbeing	FoMO impacts subjective well-being through its impact on social media intensity and social connection.
Servidio, R. et al. (2021) [31]	MEV	Narcissism; psychopathy; machiavellianism; PSU	FoMO partially mediated the association between narcissism and PSU.

Table 1 was compiled by the authors from the literature. IV indicates independent variable; DV indicates independent variable; MOV indicates moderator variable; MEV indicates mediator variable.

**Table 2:** Representative literature of FoMO-related marketing research.

Author(s)/Year	FoMO as Variable	Variables Related to FoMO	Findings
Çelik, I. K. et al. (2019) [41]	IV	Impulse buying; post-purchase regret	FoMO in sales increases impulse buying, and impulse buying increases post-purchase regret.
Kang, I. et al. (2019) [42]	IV	Conformity consumption; evaluation processes	The stability and attention caused by FoMO has led to an excessive submissiveness to the consumption of culture-related brands.
Hodkinson, C. (2019) [43]	Type of appeals	Participation decision, post-decision and emotional response	A 2 × 2 FoMO-appeal classification scheme comprising commercial vs. noncommercial and personal vs. impersonal.
Hayran, C. et al. (2020) [44]	DV	Loyalty, redo intentions; job satisfaction	FoMO reduced immediate satisfaction and a subsequent intention to repeat the experience.
Kim, J.et al. (2020)	IV MOV	Intrinsic constrains; extrinsic constraints; behavioral intention	FoMO poses a threat to loyalty by decreasing one’s intentions to repeat a current experience.
Kang, I. et al. (2020) [45]	MOV	Emotional needs; social-identity needs; brand involvement; informational/normative herd behavior	For psychological comfort, high FoMO consumers may be prone to develop high brand engagement, leading to their collective consumption of certain luxury brands.
Good, M. C. et al. (2020&2021) [46,47]	IV	Self-enhancement; anticipated expense regret; purchase likelihood	FoMO-laden appeals can influence consumers’ purchase intentions, can strengthen purchase intentions or weaken purchase intentions.
Karimkhan, F. et al. (2021) [48]	DV	Collectivism; individualism; ethnic identity	Collectivism and ethnic identity appear to be strongly correlated with FoMO.
Neumann, D. et al. (2021) [49]	IV	Attitude formation; information processing; Instagram participation	High (vs. low) FoMO expresses more favorable views of online products after being exposed to Instagram content.

Table 2 was compiled by the authors from the literature. IV indicates independent variable; DV indicates independent variable; MOV indicates moderator variable; MEV indicates mediator variable.

**Table 3:** Descriptive statistics.

Measure	Items	Frequency	Percentage
Gender	Male	120	32.79%
	Female	246	67.21%
Age	20 and below	68	18.58%
	>20 and ≤30	290	79.23%
	>30 and ≤40	6	1.64%
	41 and above	2	0.55%
Education	High School and below	2	0.55%
	Undergraduate	251	68.58%
	Postgraduate and above	113	30.87%

Sample *n* = 366; data analysis was conducted using IBM SPSS 21.0 software.

**Table 4:** Research variables and correlations.

	IT	CUSCP	II	PT	FoMO	SE	IS	PA	CB
IT	1								
CUSCP	0.791 *	1							
II	0.688 *	0.713 *	1						
PT	0.012	−0.095	0.005	1					
FoMO	0.597 **	0.903 *	0.611 *	0.015	1				
SE	0.76 *	0.96 *	0.684 **	−0.091	0.867 **	1			
IS	0.643 *	0.813 *	0.579	−0.077	0.734	0.78 **	1		
PA	−0.155	−0.196 *	−0.14	0.019	−0.177	−0.189	−0.16	1	
CB	0.461 **	0.582 **	0.415 *	−0.055	0.526 *	0.559 *	0.473	−0.114	1

** *p* < 0.01; * *p* < 0.05, *n* = 366; IT indicates influencer trait; CUSCP indicates continued use of social commerce platform; II indicates informational incentives; PT indicates personal trait; FoMO indicates fear of missing out; SE indicates social engagement; IS indicates information support; PA indicates psychological anxiety; CB indicates compulsive buying.

**Table 5:** Reliability analysis.

Constructs	No. of Items	Alpha	AVE	Loadings
Influencer traits	4	0.858	0.70	IT1(0.834) IT2(0.831)IT3(0.835)IT4(0.849)
Informational Incentives	4	0.818	0.65	II1(0.767)II2(0.809)II3(0.831)II4(0.816)
Personal traits	4	0.893	0.76	MA1(0.87)MA2(0.855)MA3(0.853)MA4(0.904)
Continued use of SCPs	3	0.854	0.77	CU1(0.893)CU2(0.904)CU3(0.843)
FoMO	4	0.883	0.74	FM1(0.843)FM2(0.842)FM3(0.863)FM4(0.896)
Social engagement	4	0.811	0.64	SE1(0.779)SE2(0.847)SE3(0.859)SE4(0.702)
Information support	3	0.814	0.73	IS1(0.823)IS2(0.868)IS3(0.874)
Psychological anxiety	4	0.931	0.83	PA1(0.867)PA2(0.916)PA3(0.924)PA4(0.933)
Compulsive buying	3	0.853	0.77	CB1(0.888)CB2(0.892)CB3(0.858)

**Table 6:** Model fitting index.

Index	χ^2^/df	GFI	RMSEA	NFI	IFI	CFI
Result	3.688	0.733	0.086	0.767	0.819	0.818

**Table 7:** Path analysis of basic model.

Path	Coefficient	S.E.	C.R.	P
IT- > CUSCP	0.287 ***	0.04	7.134	0.000
II- > CUSCP	0.36 ***	0.041	8.709	0.000
PT- > CUSCP	0.007	0.025	0.28	0.779
FoMO- > CUSCP	0.848 ***	0.091	9.322	0.000
II- > FoMO	0.375 ***	0.048	7.77	0.000
CUSCP- > SE	0.871 ***	0.076	11.413	0.000
CUSCP- > IS	0.725 ***	0.073	9.921	0.000
CUSCP- > PA	0.172 *	0.079	2.183	0.029
CUSCP- > CB	0.884 ***	0.09	9.847	0.000

*** *p* < 0.001; * *p* < 0.05, *n* = 366; IT indicates influencer trait; CUSCP indicates continued use of social commerce platform; II indicates informational incentives; PT indicates personal trait; FoMO indicates fear of missing out; SE indicates social engagement; IS indicates information support; PA indicates psychological anxiety; CB indicates compulsive buying.

**Table 8:** Path analysis of mediation model.

Path	Coefficient	S.E.	C.R.	P
II- > CUSCP	0.295 ***	0.038	8.064	0.000
II- > FoMO	0.362 ***	0.046	7.150	0.000
FoMO- > CUSCP	0.874 **	0.063	9.632	0.000

*** *p* < 0.001; ** *p* < 0.01, *n* = 366; CUSCP indicates continued use of social commerce platform; II indicates informational incentives; FoMO indicates fear of missing out.

## Data Availability

Not applicable.

## Appendix A

**Table A1:** Instrument and measurement properties.

		M	SD	Loading
Influencer traits [6,50]				
IT1	Social commerce platform influencers have rich buying and using experience.	4.86	1.21	0.834
IT2	Social commerce platform influencers have professional knowledge.	4.92	1.19	0.831
IT3	Social commerce platform influencers can actively respond to questions raised by fans.	4.86	1.02	0.835
IT4	Social commerce platform influencers are enthusiastic and responsible in responding to fan questions.	4.66	1.24	0.849
Informational incentives [57]				
II1	The product information on social commerce platforms is very detailed and diverse.	5.01	1.12	0.767
II2	The product information pushed by the social commerce platform can make me interested.	4.90	1.13	0.809
II3	Price discounts on social commerce platforms make products feel affordable.	4.90	1.36	0.831
II4	The panic-buying activities on social commerce platforms made me feel like saving money.	4.82	1.36	0.816
Materialism [106,107,108]				
MA1	If I can buy more things, I will feel that I am happier.	3.41	1.62	0.870
MA2	Shopping in a social business environment can bring me a lot of happiness.	3.13	1.52	0.855
MA3	I am very upset that I can’t afford what I want.	3.38	1.64	0.853
MA4	Acquiring material and wealth is one of the important achievements of my life.	3.30	1.54	0.904
Continued use of SCPs				
CU1	In the future, I plan to continue to use this social commerce platform.	4.97	1.23	0.893
CU2	In the future, I prefer to continue to use this social commerce platform instead of other platforms.	4.80	1.24	0.904
CU3	In the future, I will increase the frequency of using this social commerce platform.	4.68	1.23	0.843
FoMO [27,41,109,110]				
FM1	I often subconsciously open the social commerce platform to view the content.	4.75	1.38	0.843
FM2	I will frequently open the social commerce platform to obtain product information and so on.	4.65	1.44	0.842
FM3	If I do not open the social commerce platform for a period of time, I will worry about missing important information.	4.27	1.58	0.863
FM4	If I do not open the social commerce platform for a period of time, I am worried that I will miss the opportunity to obtain information.	4.28	1.59	0.896
Social engagement [36]				
SE1	The social commerce platform has influencers that I follow.	4.83	1.37	0.779
SE2	I will share ideas on social business platforms.	4.08	1.57	0.847
SE3	I will share information about products on social commerce platforms.	4.33	1.59	0.859
SE4	On social business platforms, I interact with influencers I follow.	4.77	1.44	0.702
Information support [75,86]				
IS1	This social commerce platform has improved my efficiency in obtaining information.	4.75	1.36	0.823
IS2	Frequent use of this social commerce platform allows me to obtain more information.	4.50	1.26	0.868
IS3	Frequent use of this social commerce platform has enriched my knowledge.	4.76	1.25	0.874
Psychological anxiety [37,39,60]				
PA1	I will be distressed because I cannot control my frequent use of the social commerce platform.	4.37	1.58	0.867
PA2	I will regret that frequent use of this social commerce platform reduces my learning efficiency.	4.43	1.56	0.916
PA3	I will be depressed because frequently opening the social commerce platform reduces my concentration.	4.46	1.64	0.924
PA4	I will be annoyed by the time consumed by frequently opening the social commerce platform.	4.78	1.62	0.933
Compulsive buying [79,82,87]				
CB1	Most of my daily necessities are purchased online.	4.14	1.67	0.888
CB2	Others may think I am a shopaholic.	4.10	1.65	0.892
CB3	There is an unopened package in my living area.	4.61	1.52	0.858
